# A Computational Study on Altered Theta-Gamma Coupling during Learning and Phase Coding

**DOI:** 10.1371/journal.pone.0036472

**Published:** 2012-06-21

**Authors:** Xuejuan Zhang, Keith M. Kendrick, Haifu Zhou, Yang Zhan, Jianfeng Feng

**Affiliations:** 1 Mathematical Department, Zhejiang Normal University, Jinhua, P.R. China; 2 Key Laboratory for Neuroinformation, Social Cognition and Affective Neuroscience Group, School of Life Sciences and Technology, University of Electronic Science and Technology of China, Chengdu, P.R. China; 3 Mouse Biology Unit, European Molecular Biology Laboratory, Monterotondo, Italy; 4 Center for Computational System Biology, Fudan University, Shanghai, P.R. China; 5 EMBL-EBI, Wellcome Trust Genome Campus, Hinxton, Cambridge, United Kingdom; 6 Department of Computer Science, Warwick University, Coventry, United Kingdom; University of Michigan, United States of America

## Abstract

There is considerable interest in the role of coupling between theta and gamma oscillations in the brain in the context of learning and memory. Here we have used a neural network model which is capable of producing coupling of theta phase to gamma amplitude firstly to explore its ability to reproduce reported learning changes and secondly to memory-span and phase coding effects. The spiking neural network incorporates two kinetically different GABA_A_ receptor-mediated currents to generate both theta and gamma rhythms and we have found that by selective alteration of both NMDA receptors and GABA_A,slow_ receptors it can reproduce learning-related changes in the strength of coupling between theta and gamma either with or without coincident changes in theta amplitude. When the model was used to explore the relationship between theta and gamma oscillations, working memory capacity and phase coding it showed that the potential storage capacity of short term memories, in terms of nested gamma-subcycles, coincides with the maximal theta power. Increasing theta power is also related to the precision of theta phase which functions as a potential timing clock for neuronal firing in the cortex or hippocampus.

## Introduction

The roles of different brain oscillatory rhythms, either alone or in combination, in controlling learning and memory functions have been the subject of intensive investigation and speculation. Local field potential (LFP) recordings in the hippocampus have shown that low frequency theta oscillations (4–8 Hz) are important in carrying information about memory processes [1], [2] and function to decreasing reaction times in decision making tasks [3]. Recording studies in the CA1 region of the hippocampus have also shown that both synaptic plasticity and the strength of inputs vary systematically with ongoing theta oscillations [4], [5]. On the other hand, high frequency oscillations such as gamma waves (30–80 Hz) can provide tighter co-ordinated control than those in low frequency ranges [6]. EEG and MEG as well as LFP recordings have revealed that synchronous firing of a group of neurons in visual processing is associated with binding problem in which gamma synchronization can combine features in a visual scene to form a coherent percept [7], [8]. Modulation of oscillatory synchronisation can also lead to the increase in synaptic gain at postsynaptic target sites thereby potentiating responses to learned stimuli [8], [9].

Both low and high frequency oscillations occur in many brain regions [10] and recent interest has focused on how these can be coupled and what the functional consequences of such coupling might be. With the development of mathematical tools such as Bayesian network and Granger causality analysis [10], several cross-frequency interactions have been observed, e.g. n : m amplitude-independent phase coupling [11], and the phase of slow frequency wave interacts with the amplitude of fast rhythm [12], [13]. Cross-frequency coupling (CFC) of theta phase with gamma amplitude has recently been shown to strengthen significantly as a function of learning both in the inferior temporal cortex (IT) following a visual face-discrimination task [14] and also in the hippocampus during an item-context association task [15]. The change in coupling strength also correlated positively with behavioral performance. However, while in the IT changes in coupling strength occurred in conjunction with increased theta power [14], although they appeared not to be causally linked, in the hippocampus they occurred without theta power changes [15].

Another potential functional role of theta-gamma coupling may also relate to short term memory and its capacity. In 1956, Miller first provided evidence that people can only hold around 7±2 items in a variety of short-term memory (STM) tasks [16]. It has subsequently been proposed that this capacity limit on STM storage can be explained by a multiplexing mechanism based on coupled theta and gamma oscillations [17]. If individual memory items, for instance a sequence of words, are stored in separate high frequency (gamma) subcycles coupled to a low frequency (theta) oscillation, then only around 7±2 gamma sub-cycles can occur in each theta cycle corresponding to short term memory (i.e. one memory per sub-cycle) [17]. A recent study in humans has also shown that there is a significant correspondence between the number of gamma-subcycles nested on a theta wave and actual individual short-term memory capacity [18]. Furthermore, slow NMDA receptors were found to account for recalling these stored memories at the gamma frequency range [18]. The theta wave in the neuronal networks proposed by Lisman and Jensen [17]–[19] was driven by an external input. However it has been demonstrated that there are two forms of GABA_A_ receptor-mediated inhibitory currents (slow and fast) in hippocampus [20], [21] which can generate the simultaneous occurrence of both slow and fast frequency oscillations. Recently, GABA_A_ slow inhibitory postsynaptic currents (IPSCs) have also been observed in visual cortex [22]. All these findings suggest that control over theta and gamma power and coupling can occur within both cortical and hippocampal networks using a combination of NMDA and slow and fast GABA_A_ receptors.

Recently, we have investigated the effects of face and object discrimination learning on theta and gamma oscillations and the interactions between them in sheep IT using 64-electrode recording arrays [14]. The experiment gave two prominent results: i). From the wavelet-analyzed results of the recorded LFP data, it was found substantial theta-band activity occurring at about 300 ms after the presentation of stimulus, accompanied by a much smaller contribution from gamma-band activity in the time-dependent spectrum. ii). Following training, the amplitude of theta but not gamma was increased. Over 75% of electrodes showed significant increase of the coupling between theta phase and gamma amplitude. We have already produced a spiking neural network model based on two kinetically distinct GABA_A_ receptor-mediated currents to reproduce the above visual-discrimination learning effects on theta power and theta and gamma coupling by altering the strength of NMDA receptors [14]. However, we have not fully characterised the influence of different elements of our model or tested its efficacy for generating the different patterns of learning-evoked changes observed in the hippocampus and elsewhere. The utility of the model for investigating the relationship between theta and gamma in relation to potential memory span in short-term memory tasks has also not been established.

In this paper, we firstly carried out a detailed investigation of the contributions made by the different individual components in the model to theta and gamma oscillations. Using this knowledge we then established the most effective combinations of altered synaptic mechanisms in the model which can produce the different patterns of learning-evoked changes in theta and gamma power and theta-gamma coupling and neuronal firing that have been reported in [14]. Lastly, using the same spiking neuronal network model, we investigated its utility in demonstrating the proposed relationship between short-term memory capacity and theta/gamma dual oscillations and what parameters can increase or decrease this. Our results show that this model whether in its original all-to-all connection form or with more realistic sparse connectivity is able to reproduce different permutations of learning evoked changes primarily using a combinations of altered NMDA and GABA_A_ receptor strength. They also show that while 7±2 sub-cycles can be nested on theta waves that this can be modulated by alterations in theta amplitude and phase.

## Results and Discussion

**Figure 1:**
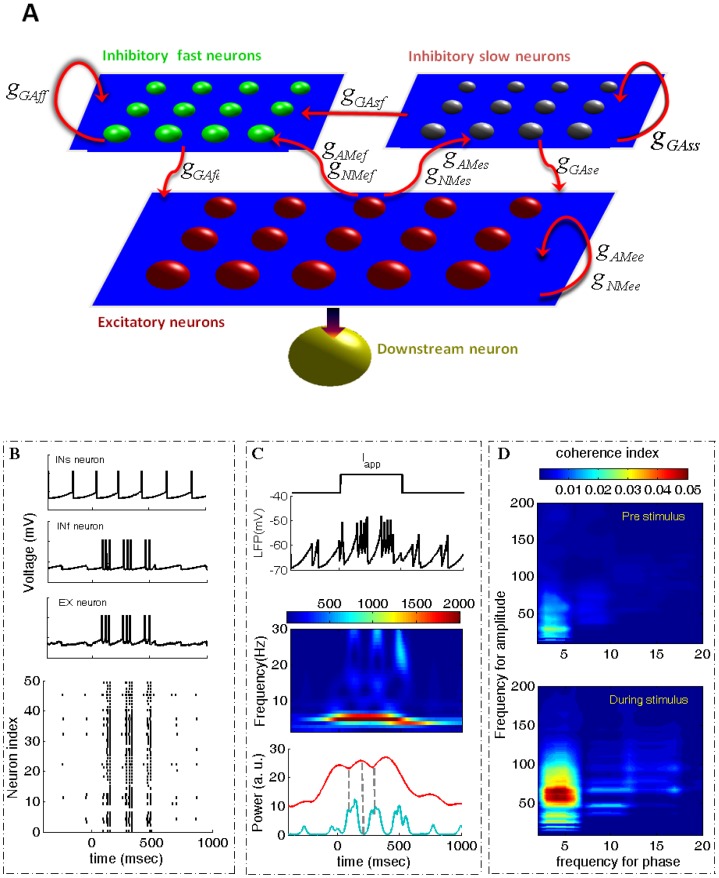
Stimulus-enhanced theta wave as well as CFC. (A) A network of 100 excitatory (EX), 50 fast inhibitory (INf) and 50 slow inhibitory neurons (INs). The outputs of EX neurons are projected to a downstream neuron. (B) The firing behaviors of single INs, INf and EX neurons. The bottom trace is the firing pattern of 50 EX neurons. (C) Input stimulus (Iapp), the LFP which is the average of membrane potentials of all EX neurons, the time-dependent power spectrum of the LFP of mean powers in the theta (red curve) and in the gamma band (blue curve). (D) Coherence of CFC between the theta phase and the gamma amplitude for the pre and during stimulus epochs.

Biophysical models for generating hippocampal theta and gamma rhythms have already been provided by Kopell et al. in [23], where it was claimed that theta nested-gamma activity is due to the h-current in the oriens-lacunosum molecular cell in the hippocampus. Instead of using a Hodgkin-Huxley type neuronal network with the h-current, here we applied a simple spiking neural network based on two kinetically distinct GABA_A_ receptor-mediated currents to explore the synaptic mechanism of learning-evoked changes in theta amplitude and theta-gamma coupling. A schematic showing the neural network model is given in Figure 1A. Here only 100 excitatory (EX) neurons, 50 inhibitory fast (INf) neurons and 50 inhibitory slow (INs) neurons with all-to-all or sparse connections are considered. Each cell, receives AMPA and NMDA receptor mediated currents from pyramidal cells and GABA_A_ receptor mediated currents from INf and INs neurons. The weight and direction of the connections are shown in Figure 1A. For example, 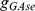
represents the connection from INs neuron to EX neuron mediated by GABA_A_ receptors, 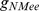 represents the recurrent connection among EX neurons mediated by NMDA receptors, etc. For detailed modeling equations of the network, see the methods section. To mimic a typical visual-evoked response lasting 300 ms we applied a transient current pulse to represent the stimulus, with intensity corresponding to stimulus strength and the transient time corresponding to stimulus duration.

Figures 1B, 1C and 1D respectively illustrate the effects of an applied stimulus lasting 300 ms in the model on the firing of the slow and fast inhibitory and EX neurons, on the local field potential (LFP), power spectrum and theta and gamma amplitude and on the strength of coupling between theta phase and gamma amplitude. This mimics multi-unit neuronal activity (MUA spikes) and the averaged field potential (LFP) recorded in the animal’s IT cortex in the presence of a transient object representation.

### The Importance of Fast and Slow Inhibitory Neuron Connections for Generating Theta and Gamma Oscillations, their Coupling and Neuronal Firing

**Figure 2:**
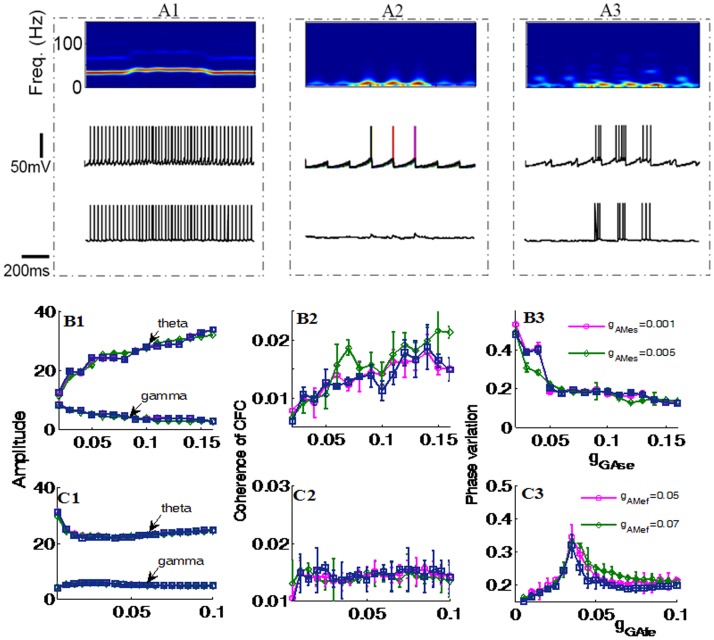
Coordinated regulation of GABA_A,fast_ and GABA_A,slow_ currents is the key for generating theta-nested gamma oscillations. (A) Three different response behaviors of the network to a stimulus: (A1) Only gamma rhythm (by blocking INs→EX connection); (A2) only theta rhythm (by blocking INf→EX connections); (A3) theta-nested gamma rhythm (in the presence of both INs→EX and INf→EX connection). The three traces from the upper to the bottom are the time-frequency power spectrum, the firing behaviors of EX cells and the firing behavior of a downstream neuron, respectively. (B-C) Effects of increasing g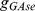 and 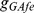 on the theta and gamma amplitudes, the coherence of cross-frequency coupling, the tightness of theta phase.

In order to establish the key contributions of the slow (INs) and fast (INf) inhibitory neuron connections in the network for influencing theta and gamma power and coupling we compared the effects of three different network configurations upon them (see Figure 2A).

Figure 2A1 shows that if the connection from the INs cells to the EX cells is blocked (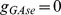) EX cells only exhibit gamma oscillations. Contrarily, when the connection from the INf inter-neurons is minimal (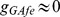) and that from the INs ones (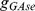) is strong, it is seen in Figure 2A2 that theta power is enhanced but no neuronal spikes fire in the gamma frequency range and as a result the downstream neuron becomes silent. Only when both 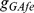 and 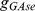 are functionally modulated, then the EX cells exhibit theta-nested gamma oscillations and the magnitude of the averaged LFP is significantly increased in response to the stimulus (see Figure 2A3). In this case, the power spectrum is highly concentrated in the theta band and increases when the stimulus is applied. Moreover, compared with the case in Figure 2A2, here both the EX and downstream neurons are more active during the period when the stimulus is on. Hence both INf→EX and INs→EX connections are required to achieve the presence of both theta and gamma and oscillations. An optimal coupling between them is necessary for both EX and downstream neurons to respond strongly and selectively during a stimulus.

To explore more fully the effects of these two inhibitory synaptic couplings on the behavior of the network we plotted theta and gamma amplitudes, the coherence of CFC and theta phase variation as a function of increases in either 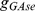 or 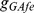 strengths. Effects of increasing the reciprocal connection strength were also plotted by increasing the strength of AMPA receptor (*g*_AMes_ and *g*_AMef_). In Figures 2B and C, respectively it can be seen that across a range of 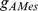 values, as 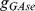 is strengthened then gamma amplitude exhibits a monotonic decrease while theta amplitude progressively increases. The coherence of CFC also increases progressively a s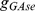 is strengthened and the variation of theta phase decreases. Increasing *g*_AMes_ has very little effect. This tells us that strengthening the connection from INs neurons to EX neurons not only increases theta amplitude, but also coupling between theta phase and gamma amplitude. There is also tighter regulation of the timing of theta phase. An explanation for these findings is that with the increase in 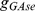, the EX neurons are more and more tightly controlled by the theta-band oscillation from the INs neurons. Since the synaptic inputs from INs neurons are inhibitory, the firing rate decreases with the increase in 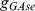, resulting in a reduction in the number of nested spikes in each theta cycle and a corresponding decrease in gamma amplitude.

Figure 2C also shows that when the INf→EX connection is strengthened theta amplitude gradually decreases at first but, after reaching a minimum, starts to increase slightly again as 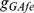 is further strengthened. Gamma amplitude on the other hand shows the opposite pattern slightly increasing to begin with and then decreasing again as 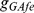 is strengthened progressively. The strength of theta-gamma coupling remains fairly constant and theta-phase variation is initially sharply increased and then slowly reduces in a similar pattern to that of theta amplitude. These observations show that when 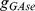 is not strong and the INf→EX connection is weak, then there are very high frequency bursting oscillations nested in each theta cycle so that gamma amplitude is weak while theta amplitude is strong. With the increase strength of 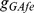, the nested high frequency oscillations gradually shift to oscillations in the gamma band and thus gamma amplitude increases while theta amplitude decreases. Since the synaptic inputs from INf to EX neurons are also inhibitory, further increasing 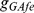 will eventually shut down the gamma-band oscillations although theta-band (subthreshold) oscillations are always present. Thus after a critical value of 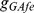, gamma amplitude decreases while theta amplitude increases.

**Figure 3:**
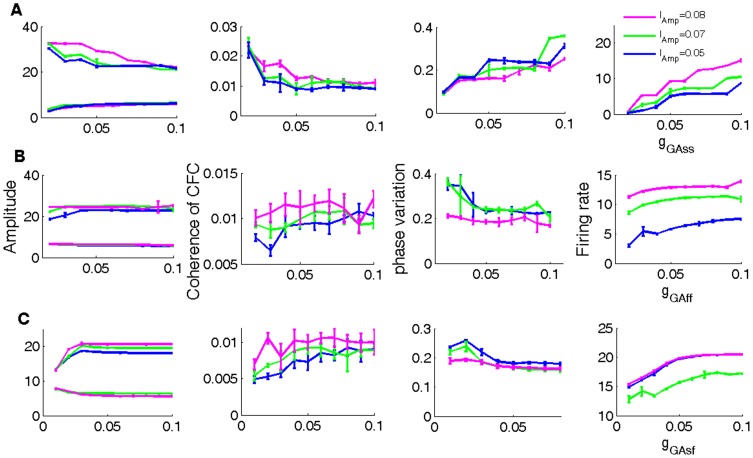
Effects of modulating GABA conductances between and within inhibitory cells. The amplitudes of theta and gamma oscillations, the coherence of CFC, theta phase variation and the firing of excitatory neurons as a function of changes in the strength of (A) *g_GAss_*_’_ (B*) g_Gff_* (C) *g_GAsf_* are shown.

Figure 3 shows the effects of altering the strength of connections within (*g*_GAss_ in INs and *g*_Gaff_ in INf) and between inhibitory (*g*_GAsf_ from INs to INf) neurons. The three connections all contribute to increased firing rate but they have different effects on the theta amplitude and theta-gamma coupling: The connection within INs neuron *g*_GAss_ decreases theta amplitude as well as the coherence of CFC between theta and gamma, while the connection *g*_GAsf_ is responsible for increasing theta amplitude and theta-gamma coupling (although increasing the strength of this connection too much tends to saturate these two quantities). Increasing the connection within INf neurons *g*_GAff_ does not have much effect on increasing either theta amplitude or theta-gamma coupling (see Fig 3B).

In summary, the simultaneous occurrence of theta and gamma oscillations requires the presence of recurrent couplings in both INf and INs neurons, and a delicate balance between the INs→EX and INf→EX connections. To increase theta amplitude as well as the coherence of CFC between the two rhythms, the INs→EX connection should be relatively strong, while the INf→EX is required to be relatively weak.

### Effects of Excitatory Neuron Connections on Altering Theta and Gamma Oscillations, their Coupling and Neuronal Firing

**Figure 4:**
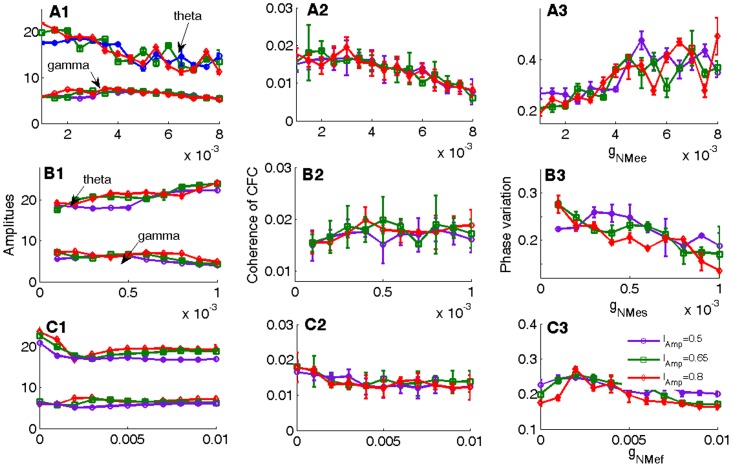
Effects of modulating NMDA conductances from excitatory to inhibitory neurons. The amplitudes of theta and gamma oscillations, the coherence of CFC, the phase variation vs.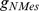 and 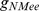 are depicted, respectively. (A) shows the effect of increasing 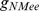, (B) shows the effect of increasing 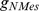, (C) shows the effect of increasing 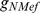.

We found that just increasing the conductance of the NMDA receptors between and within EX cells (*g*_NMee_) increased their firing rate and that of the downstream neuron. Coupling between theta and gamma was initially stable but then was reduced, whereas theta phase variation progressively increased (see Figure 4A). On the other hand increasing NMDA receptor conductance between EX and INs neurons (*g*_NMes_) increased theta amplitude, but slightly decreased gamma amplitude. Theta-gamma coupling increased progressively in strength whereas theta phase variation decreased.

Increasing the coupling strength between EX and INf neurons (*g*_NMef_) has the effect of reducing the firing frequency of EX and the downstream neuron and theta amplitude. It also reduces theta-gamma coupling and increases theta phase variation while having no effect on gamma amplitude (see Figure 4).

### Effects of Sparsening Network Connections

We chose at the outset for simplicity to use an all to all connection design in our network. However, to show that the results obtained are not entirely dependent upon this design we also investigated if they could be replicated by a progressively sparsened network, which would perhaps be more representative of normal physiological neural networks. The same numbers of neurons were included in the network and sparseness was realized by randomly assigning the coupling between neurons. In this case the probability that a pair of neurons are connected in either direction is p = 0.8. Results shown in Text S1 confirmed that the sparsened network produced similar changes in theta and gamma parameters, including theta-gamma coupling (see Figure S1).

Although such a coupling probability between neurons is still far from estimating real neuronal networks, larger network sizes can compensate for sparse connectivity [24], [25]. It has been previously reported that in network of 10^6^ neurons with 5% sparseness and an average rate of 5 spikes^−1^ received by a neuron, then simulating a network of 10^5^ neurons, the sparseness could increase to 20% and average rate of 12.5 spikes^−1^ to obtain the same afferent spike statistics [24]. In our model, numerical simulation shows that when the network size increases to *N*_EX_ = 200, *N*_INf_ = 100, *N*_INs_ = 100, the coupling probability can be reduced to p = 0.6 and all results still hold true (see Figure S2).

### Learning Effects on Theta Amplitude and Theta-gamma Coupling Optimally Require Coordinated Regulation of NMDA and GABA_A,slow_ Receptors

Experimental recordings in sheep IT cortex have revealed that after learning, theta amplitude and the theta/gamma ratio as well as the strength of theta-gamma coupling are enhanced whereas gamma amplitude remains unchanged. The proportionate changes in these parameters were positively correlated with actual discriminatory performance [14]. On the other hand in the dorsal hippocampus of rats theta-gamma coupling is increased in rats after they learned to associate items with their spatial context but without any increase in theta amplitude and again the strength of theta–gamma coupling was directly correlated with the increase in performance accuracy during learning sessions [15]. We used our neural network model to investigate whether it could reproduce both of these outcomes.

**Figure 5:**
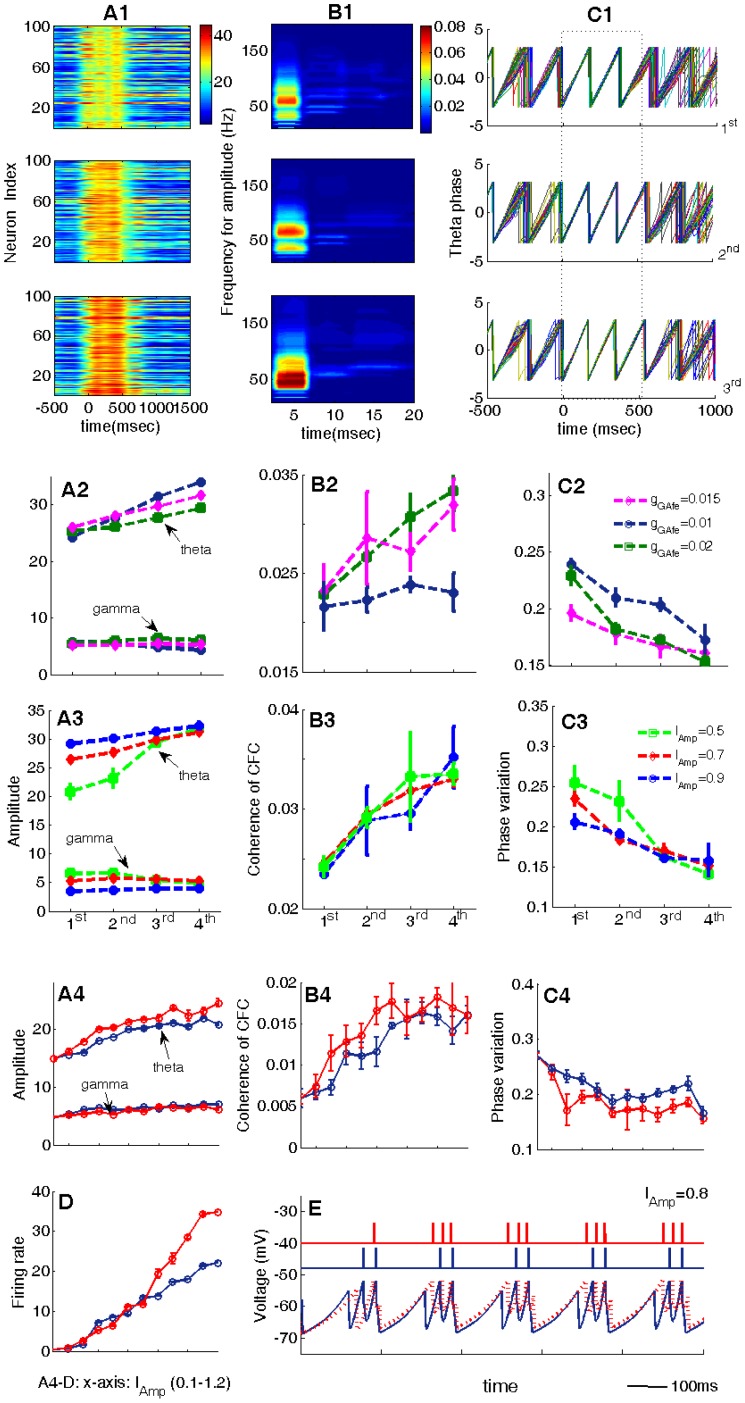
Effect of coordinately regulating synaptic gains of NMDA and GABA_A,slow_ to simulate learning effects. (A1–A3) Theta amplitude. (B1–B3) The coherence of CFC. (C1–C3) The temporal dynamics of theta phase. The stimulus is applied during 0–500 ms. To mimic different learning stages, we set NMDA receptor mediated conductances 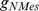, 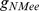 and the GABA_A,slow_ mediated conductance 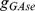 as: 0.0001, 0.001, 0.05 (1st), 0.00025, 0.0025,0.06 (2nd), 0.00035 and 0.004, 0.07 (3rd), 0.00045, 0.005, 0.08(4th). The values of 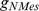 and 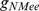 in the three panels in A1–C1 are corresponding to the three marked points (1^st^, 2^nd^ and 3^rd^) in A2–C3. The values of other parameters are stated in Table 1. (A4–C4) Variations of theta and gamma amplitudes, the coherence of cross-frequency coupling and the phase variation with the increase of 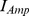. (D) The firing rates of a downstream neuron vs.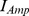. (E) Comparison of the membrane potentials of single EX neurons before and after learning. In (C) the blue curve where 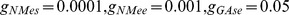 represents before learning, while the red one where 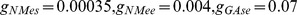 represents after learning.

Firstly we investigated the role of NMDA and GABA_A_ receptors in mediating changes where both theta amplitude and theta-gamma coherence are altered. This confirmed that altering the strength of recurrent coupling of NMDA receptors in EX neurons and that between EX neurons and INs neurons could reproduce the findings in IT as we have previously reported [14]. However, it was found that only moderate increases in theta amplitude could be produced by just using changes in NMDA receptors (both *g*_NMee_ and *g*_NMes_). We found that a more robust and greater range of increased theta amplitude in conjunction with strengthened theta-gamma coupling could be produced by increasing both the strength of the NMDA receptors and that of the GABA_A_ receptors between the INs and EX ones (*g_GAse_*) (see Figure 5). This had the effect of both increasing EX and downstream neuron activity. Increasing the conductance 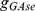 alone results in decreased firing by EX neurons leading to a decreased firing rate by the downstream one. However, the decreased firing rate of a downstream neuron caused by the increase of 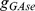 can be compensated for by moderately increasing the conductance 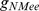. Interestingly it has also recently been proposed that homeostatic synaptic plasticity may optimally involve both changes in glutamatergic and GABAergic transmission [26]. The remaining parameters in the model do not appear to play an important role in generating such learning effects as those observed by coordinately regulating NMDA and G_ABAA, slow_ receptors. Indeed, Figures 2, 3 and 4 show that altering the couplings g_GAfe_, g_GAff_, g_GAss_, g_NMef_, g_NMee_ do not result in increased theta amplitude, and the parameter range of *g_GAsf_* for increasing theta amplitude is restricted to a very small range.

**Table 1:** Values of parameters.

Variable	Definition	Value	Range
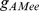	EX→EX connection mediated by AMPA receptors	0.03	0.01–0.05
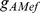	EX→INf connection mediated by AMPA receptors	0.03	0.02–0.08
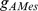	EX→INs connection mediated by AMPA receptors	0.001	0.0001–0.01
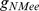	EX→EX connection mediated by NMDA receptors	0.001	0.0001–0.008
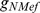	EX→INf connection mediated by NMDA receptors	0.001	0.0001–0.005
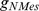	EX→INs connection mediated by NMDA receptors	0.0001	0.0001–0.0005
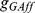	INf→INf connection mediated by GABA receptors	0.05	0.01–0.08
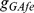	INf→EX connection mediated by GABA receptors	0.015	0.005–0.05
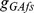	INf→INs connection mediated by GABA receptors	0.00	0.0–0.05
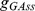	INf→INf connection mediated by GABA receptors	0.08	0.007–0.05
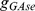	INf→EX connection mediated by GABA receptors	0.06	0.02–0.1
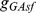	INf→INs connection mediated by GABA receptors	0.04	0.02–0.08
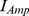	The stimulus amplitude	0.8	0.5–1.5

We used the network model to further explore the corresponding dynamic mechanism behind all these learning-altered changes. Numerical simulations show that increasing 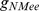 induces gamma waves to become more and more shallowly nested on the theta wave (Figure 5E), but at the same time it increases the number of the nested spikes per theta cycle (i.e., increasing the firing rate of EX cells). The former situation is favorable for increasing theta power, while the later plays an opposite role by redistributing the power from the slow frequency band to the high frequency band. The distribution of power in the two frequency bands depends on the competition between the number and shape (deep or shallow) of the nested gamma subcycles. If only the conductance 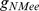 is increased, the effect of increasing the firing rate but decreasing theta amplitude may compete with that of increasing theta amplitude by enhancing the shallowness of the nested gamma. Thus it is difficult to produce all potential learning effects involving increased theta amplitude by only increasing 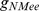. However, the GABA_A,slow_ receptor-mediated synaptic currents from the INs neurons to the EX neurons, which are comparable to the time scale of the theta range oscillations, in turn give feedback to EX neurons via the INs→EX connection. With the increase of the conductance 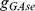, the EX neurons are more and more tightly controlled by the theta-band oscillations, but at the cost of decreasing the firing rate. Thus it is necessary to modulate the conductance of NMDA receptors together with the conductance of GABA_A_ receptors to enhance both the effects in frequency (theta power and the phase-to-amplitude modulation) and time domains (the concentration of the theta-band phases and the firing rate of a downstream neuron), as observed in Figures 5A4–C4 and Figure 5D.

Finally we confirmed that we could produce the same learning outcomes using a sparsened as opposed to an all-to-all version of our network for both the situation where only conductances of NMDA receptor are altered (see Figure S3) or where both NMDA excitatory and GABA inhibitory connections are altered (see Figure S4). As with the all-to-all network the combined changes in NMDA and GABA synaptic strengths produced the most robust effect.

In the second learning scenario involving the hippocampus it was reported that, unlike in the IT [14], increased theta and gamma coupling strength occurred without a corresponding increase in theta amplitude [15]. It can be seen from Figure 5 that this cannot be reproduced by changes in 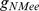 together with 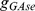 since across the range of stimulus strengths applied theta amplitude is always increased as well as the strength of theta-gamma coupling. However, it can be seen from Figure 2C that by slightly increasing *g*_GAfe_ from a small value (from 0.005 to 0.02) theta amplitude decreases but the strength of theta gamma coupling is increased. It can further be seen in Figures 3B and C that increasing the couplings *g*_GAff_ and *g*_GAsf_ results in increased theta amplitude as well as the theta-gamma coupling. Thus we can balance the decreased and increased effects on theta amplitude produced by increasing the coupling strengths of both *g*_GAfe_ and *g*_GAsf_ + *g*_GAff_. At the same time the changes in these three couplings can produce an increase in the coherence of CFC between theta and gamma. Figure S5 illustrates this finding and shows that increases in theta amplitude are not necessarily correlated with increases in theta-gamma coupling. Obviously the possibility that plasticity changes in these different GABA receptors are important requires experimental support and there are clearly other potential variants of coupling changes that can result in a similar learning outcome and could involve changes in NMDA as well as GABA receptors.

### Short-term Memory: Theta Amplitude, Phase Precision and Memory Capacity

**Figure 6:**
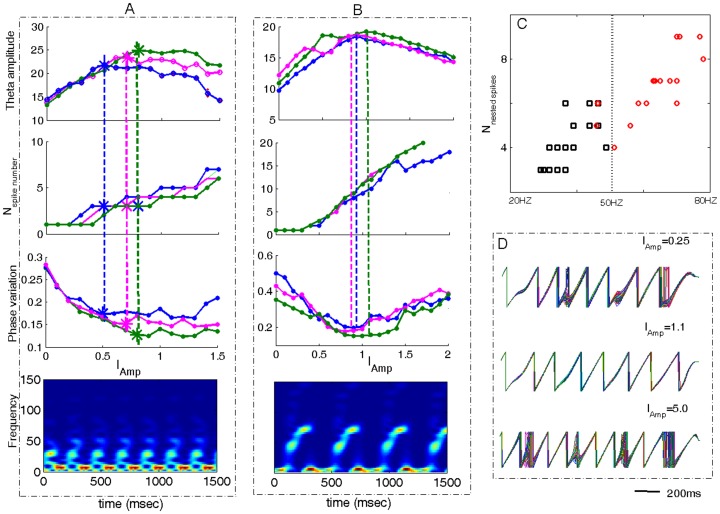
Relationship of maximal theta amplitude, the number of nested spikes per theta cycle and theta-phase concentration. (A-B) From the upper trace to the bottom trace:Theta amplitudes vs. the stimulus strength 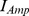, the corresponding number of nested spikes per theta cycle vs.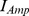, theta phase variation vs. 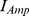, and the time-frequency spectra at 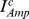 of one curve. In (A), 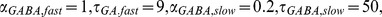 the three curves correspond to 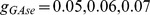. In (B), 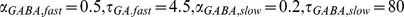, the three curves correspond to 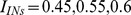. (C) The number of nested spikes per theta cycle calculated at 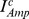 vs. the frequency of the corresponding maximal gamma power. The marked points are obtained from different curves of theta amplitude vs. 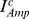. One can see that for low gamma power (20–50 Hz), around 5±2 spikes could be nested in each theta cycle, while for high gamma power (>50 Hz), around 7±2 spikes could be nested. (D) Theta phases of the EX cells for different stimulus strength. It was shown that for too weak and too strong stimulus, the theta phases of neurons are less synchronized than that of the intermediate stimulus strength.

Here we have tested the ability of our model to reproduce the proposed function of the dual oscillations in limiting the capacity of short-term memory. We first investigated the spectrum property of combined theta/gamma oscillations in the short-term memory. Figures 6A and B show that with increases in stimulus strength the amplitude of the theta-band oscillation exhibits a bell-shaped property, reaching a maximum at a critical stimulus strength 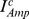. Corresponding to the peak of theta amplitude the number of nested gamma spikes per theta cycle, denoted as 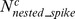, is around 3∼9 (see Figures 6A and B). Spike and gamma-sub-cycle activity are highly synchronized such that each spike corresponds to a gamma-subcycle. Interestingly, it was shown that when 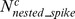 = 5±2, the frequency range of maximal gamma power at 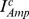 is around 30∼50 Hz (Figure 6A); and when 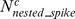 = 7±2 (Figure 6B), the frequency range of gamma power at 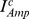 is around 50∼80 Hz.

To see whether this was a general result, we calculated different curves of theta amplitude vs. the stimulus amplitude 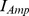, by modulating the system parameters 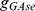 and background currents. From these curves, we counted the number of nested spikes per theta cycle at the critical stimulus amplitude 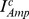 and plotted 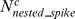 vs. the frequency corresponding to the maximal gamma amplitude in Figure 6C. It was found that 5±2 nested spikes correspond to low-frequency gamma oscillations, whereas 7±2 spikes correspond to high-frequency gamma oscillations.

Why does theta amplitude reach its maximum at around 3∼9 gamma sub-cycles per theta cycle? The reason can be understood from the counterbalance of the shape of nested gamma wave and the number of nested gamma-range spikes. When the stimulus strength 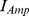 is weak, the oscillations are mainly sub-threshold and under these circumstances increasing 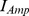 will push the membrane potentials of EX neurons closer to the threshold. As a result, the amplitudes of both theta and gamma oscillations increase. One may intuitively think that theta amplitude attains a maximum at a stimulus strength which drives the EX neurons to fire only a single spike in each theta cycle. However, this is not the case because there are sub-threshold gamma oscillations that are deeply nested in the theta wave. As the stimulus strength increases, these deeply nested gamma oscillations gradually become more and more shallowly nested. This effect plays a dominant role in increasing theta amplitude when 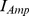 is small. But, when the stimulus strength becomes too strong, the increased number of gamma sub-cycles in each theta cycle gradually redistributes the power from theta band to gamma band, which reduces theta amplitude. The competing result of the shape vs. the number of nested gamma-range spikes produces a critical value of the stimulus strength, say 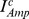, that maximizes theta amplitude.

**Figure 7:**
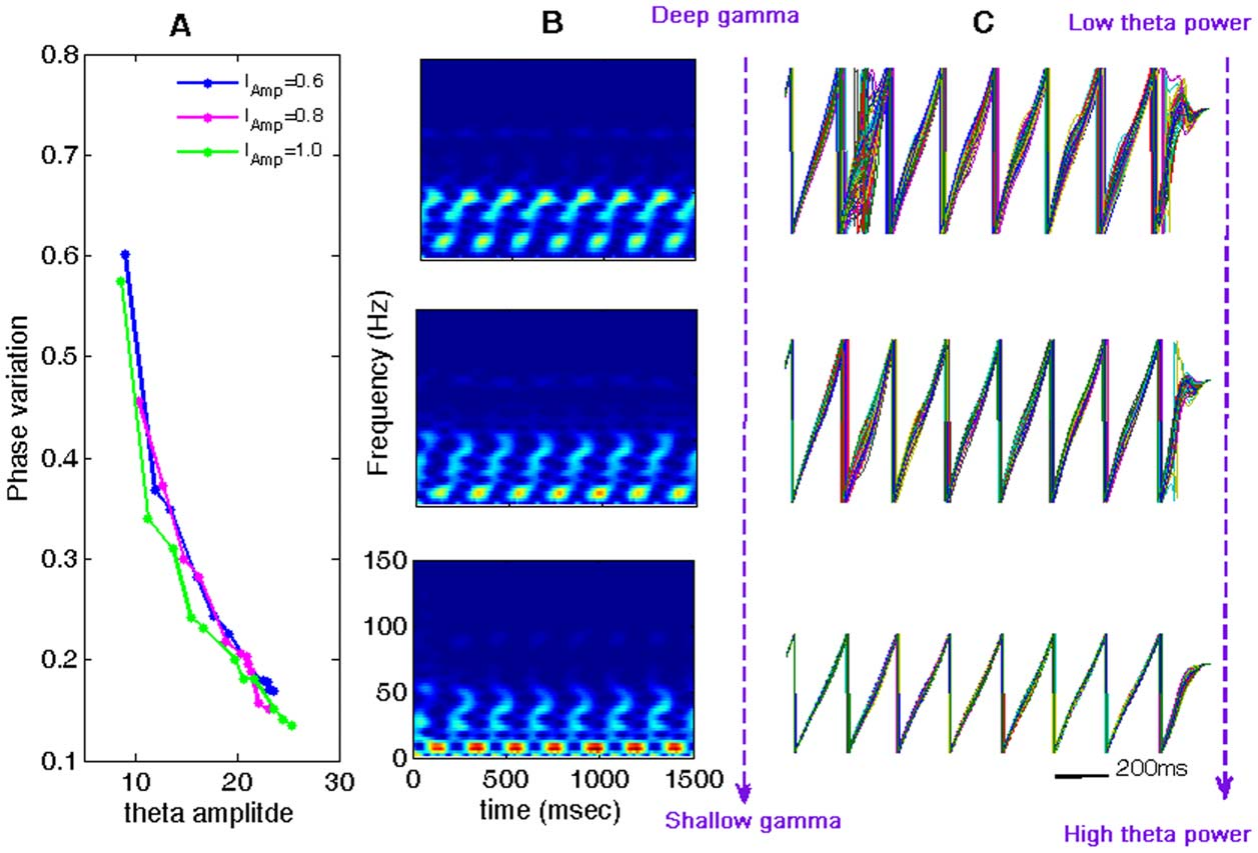
Relationship of theta amplitude, shape of nested gamma wave and the phase precision. (**A**)**.** Variation of theta phase vs. theta amplitude. The increased theta amplitude is realized by increasing the conductance 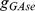. (**B**) and (**C**)**.** From the upper panel to the bottom panel are corresponding to three points chosen for low, intermediate and high theta powers in (A) for 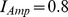. It was shown in that with the increase of theta amplitude, gamma oscillation becomes more and more shallowly nested in the theta rhythm, and meanwhile, the theta-band phases among EX neurons become more and more concentrated.

From the large body of evidence showing the involvement of both theta and gamma oscillations in working memory and in phase precession of hippocampal place cells, it appears that the phase of the theta oscillation functions as a clocking system for a neural code: phase coding. In view of the coincidence of maximal theta amplitude and storage capacity shown in Figures 6A and B, we speculated that the peak of the theta amplitude, or the optimal storage capacity, could be related to the precision of theta phase. To verify this, we applied a Hilbert transform on the membrane potentials of the EX neurons and extracted the corresponding phases of the theta waves. The variation of theta-band phases of the EX neurons was then calculated at each time point. The time-averaged value of the variations is used to measure the precision of the theta phase, and the phase variation is shown in Figures 7A and B. Interestingly, our computational results revealed that maximizing theta power corresponds to minimizing the variation of theta phases (this is in line with positive correlation between theta amplitude and theta-phase variation we have already presented in the previous sections). Figure 7D gives an illustration of the effects of stimulus strength on theta phase and from this is can be seen that for too weak or too strong stimulus strengths, the variations in theta phases are both larger than that of the intermediate stimulus strength 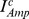.

To further show the relationship of the theta power and the phase precision, we modulated the synaptic connection from INs to EX cells. It was shown that with the increase of 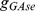, theta power increases, while the variation of theta-band phase decreases correspondingly. The variation of theta phase in EX cells vs. theta power is plotted in Figure 7A. For illustration, we selected three points (with weak, intermediate and lager theta amplitudes) in the curve of 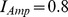 in Figure 7A, and plotted the corresponding time-frequency spectrum and theta phase in Figures 7B and C, respectively. From these it is clear that with the increase of the theta amplitude, the gamma oscillation becomes more and more shallowly nested in the theta wave, and the theta-band phase in EX cells becomes less and less variable resulting in a more accurate in phase code.

The fact that theta amplitude reaches its maximum at around 7 gamma subcycles per theta cycle also has functional significance. Biological systems are usually assumed to work in an optimal setting. Since maximizing theta power corresponds to minimizing the variation of the theta phase among neurons, it is reasonable to assume that neural networks involved in learning tend to maximize theta power during the performance of memory tasks. As an example, let us suppose that there are 7 spikes (i.e., 7 memory items) per theta cycle at 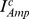 which corresponds to the maximal theta power. In performing a short term memory task with less than 7 items (for an example, 3 items: A B C), the phase variation is large since the power is weaker than the optimal one. But the phase can be improved by simply including more items (for example A A B B C C C or A B C D E F G) which drives theta power close to its maximum. However, with items larger than 7 (for an example, 10 items: A B C D E F G H I J), the theta power is lower than the maximum and phase variation is large. If one wants to improve the precision of retrieving memory under the latter circumstances then some items need to be lost. This indicates that the storage capacity of memory should correspond to the maximal theta power: one can retrieve all the memory items in the left side of 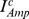, but cannot retrieve all the items in the right side of 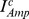. From this a reasonable assumption would be that the number of nested gamma sub-cycles which result in maximum theta amplitude represents the maximum accurate memory storage capacity. This number is consistent with the one proposed by Lisman and his colleagues from the time domain [17]–[23], [26], [27] However, in their model it was impossible to elaborate the underlying mechanism whereby theta and gamma oscillations optimally distribute their powers in processing information. In contrast, our model enables us to show how the dual oscillations may contribute to short-term memory capacity by optimally distributing their power. In the following subsection, one can further see that this capacity limit obtained from the maximal theta power also corresponds to the precision of phase coding.

It was recently pointed out that working memory maintenance in general is accompanied by increased coupling between theta phase and gamma amplitude [28]. The results shown in previous sections (Figure 2B and Figure 5B) provided a consistent conclusion that increasing theta power is accompanied by enhanced phase-to-amplitude modulation and improved precision of phase coding. Together these findings demonstrated that maximizing theta power may correspond to the best retrieval of working memory, accompanied by the strongest modulation of theta phase to gamma amplitude.

### Hippocampal Place Cells: Phase Precision and Position Reconstruction

From the results above, we concluded that the working memory capacity limit proposed by others from the time-domain information [16], [17], [29], [30], coincides with the optimal capacity obtained from the maximal theta amplitude from the frequency domain. Furthermore, the maximum theta power is achieved when theta-band phase becomes most precise. This indicates that 7±2 nested spikes per theta cycle corresponds to the most precise theta phase. This is also in agreement with what Jensen and Lisman have proposed in their work on the contribution of the theta phase to position construction from an ensemble of hippocampus place cells [31]. In their experiment, they simultaneously recorded spikes from 38 hippocampal place cells of rats which were trained to run for a food reward in a triangular maze. Spikes with inter-spike intervals in a theta period were considered and were assigned a phase for further data analysis. The first 500 s of recorded spikes were used to construct correlations of position and the firing phase of individual cells, and the last 500 s of data were used to reconstruct position from the observed spikes. The decoding error was defined as the average distance between the reconstructed position and the actual position. It was found that the best reconstruction was obtained when theta phase is more finely divided into around 7 bins (see Figures 7 A–C in [31]). In our model, we simply considered the averaged variation of theta-band phase in the each of the 100 EX cells. We found that if the number of nested spikes was smaller than 3 or larger than 9 then the theta phase variation was larger than when 7 spikes were generated. This agreement between the modeling and the experimental results shows that when too few spikes are nested in a theta cycle this is not enough to reconstruct position precisely, whereas when too many spikes are nested in a theta cycle this introduces redundant information, causing an inaccurate reconstruction. Thus in agreement with the results from Jensen and Lisman [31], we conclude that around 7 spikes per theta cycle can precisely reconstruct or retrieve memory.

### Summary

The results we have obtained from a simple spiking neuronal network show that several oscillation related phenomena can be produced by configuring a particular set of parameters: i). The network can successfully generate theta and gamma oscillations as well as coupling between theta phase and gamma amplitude. It can also do this whether in its original form where the network has an all-to-all connection configuration or where this connectivity is sparsened. We have also shown that two kinetically different GABA_A_ (GABA_A,slow_ and GABA_A,fast_) receptor-mediated currents are key in generating theta-nested gamma oscillations while the rest of parameters do not play an important in producing such oscillations although they may help shape the gamma oscillation form. ii). In either all-to-all or sparsened connection form the model can also successfully reproduce observed learning-induced changes in theta-gamma coupling in either the cortex [14] or hippocampus [15]. In the first learning scenario where both theta amplitude and theta-gamma coupling are increased (as observed in IT cortex), coordinated regulation of NMDA and GABA_A,slow_ receptors-mediated currents are shown to be the underlying synaptic mechanism. In the second learning scenario where increased theta and gamma coupling occur without a corresponding increase in theta amplitude (as observed in hippocampus), we can reproduce this phenomenon by increasing the coupling strength of both g_GAfe_ and g_GAsf_ +g_GAff_. iii). Finally the presented model could also be used to further elucidate a mechanism whereby an optimal working memory capacity of around 7 can be explained by interactions between theta and gamma coupling. Here it showed that maximal theta amplitude and synchronization occur across the network when an optimal number of 7 gamma sub-cycles are nested on each theta wave.

While our numerical results were obtained using only a small network, they could easily be extended to larger size networks. However, simply increasing the network size will destroy the established rhythm by a small network. Actually, in a sparsely connected network of excitatory and inhibitory networks, there is a very rich behavior including synchronous regular states, synchronous irregular states, asynchronous regular states as well as quench states [25]. The occurrence of these states and the transition from one to another depends crucially on the network size, the sparseness of its connections, the delay of synaptic interactions and the external inputs as well as other factors. Indeed, finite size effects on spatial and temporal aspects such as entrainment and transition synchronization are quite complex and a thorough investigation into these issues was beyond the scope of this current work. Nevertheless, we would expect the major conclusion reached here using a small network would still hold in a larger spiking network by properly scaling the probability of connections between neurons and reweighting the synaptic couplings.

## Methods

### Model

We constructed a spiking neuronal network consisting of three populations of neurons: 100 excitatory (pyramidal) neurons, 50 inhibitory fast (inter) neurons and 50 inhibitory slow (inter) neurons with all-to-all connections (see Figure 1(G)). Each set of neurons obeys an integrate-and-fire equation:
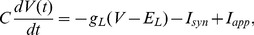
(1)where *C* is the capacitance for the neuron, 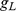 is the leaky conductance, 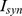 is the synaptic input from other neurons and 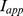 is the external input. When 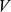 reaches a firing threshold 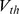, a spike is discharged and 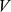 is reset to 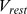 and stays there for an absolute refractory period 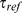. For excitatory neurons, we set 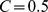 nF, 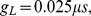 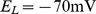 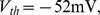 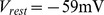 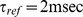 while for inhibitory neurons, we set 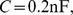 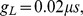 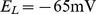 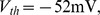 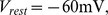 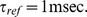


Each neuron receives AMPA and NMDA receptor-mediated currents from excitatory (EX) cells, GABA_A_ receptor-mediated currents from fast inhibitory (INf) neurons as well as slow inhibitory (INs) neurons. The gating variable 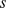 for AMPA and NMDA receptors is described by two first-order kinetics [32]:
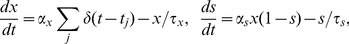
(2)where 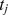 is the presynaptic spike time. We used 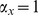
(in dimensionless), 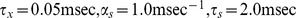 for AMPA receptors, and (in dimensionless), 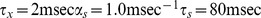 for NMDA receptors. The gating variable 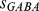 for GABA_A_ receptors obeys a simple first-order kinetics [33]:
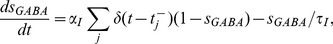
(3)where 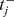 indicates the time immediately before the spike at time 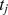. We used 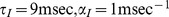 for the fast GABAA channels, and 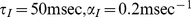 for the slow GABAA channels. The AMPA and NMDA receptors-mediated currents are given by: 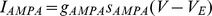, and 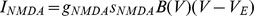, respectively, with 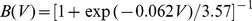. The GABAA receptor-mediated current is given by 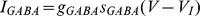. Here 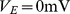,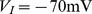.

We assumed that all neurons receive background currents all of the time. In studying learning mediated alterations of theta and gamma parameters these were set as: 0.7 (1±10%) nA for EX neurons, 0.85 nA for INf neurons and 0.6 nA for INs neurons. The stimulus was assumed to be applied to the EX neurons. The strengths of synaptic connections are given in Table 1.

### Local Field Potential

Recent report of local field potentials (LFPs) recorded in macaque IT cortex has confirmed that LFPs are selective to different stimuli [34] and carry robust information that can be used to decode the object category and identity rapidly and accurately [35]. Although it is still unclear whether the LFP is related to synaptic or ionic current, or membrane potentials [36], here we adopted the description of the LFP in the model as the average of membrane potential of the 100 EX cells in the network [37], i.e.,
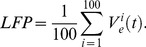
(6)


### Time-frequency Analysis

To extract more information relating to time, frequency and space, we used a wavelet transform convolving the LFP 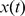 with a mother wavelet 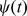 [38]:
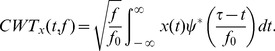
(7)Here we used a Morlet wavelet 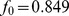 defined as



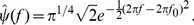
(8)We extracted the amplitudes (or powers) of the wavelet transform at 4–8 Hz and averaged them across this frequency band. For the gamma band, amplitudes in the 30–70 Hz frequency range were averaged. We therefore have time-dependent mean amplitudes (or powers) for theta and gamma rhythms, as we shown in the bottom of Figure1C. To quantify the learning-related changes in theta and gamma amplitudes, we further averaged the above time-dependent mean amplitudes over the time and got a quantity for averaged theta amplitude as well as a quantity for gamma amplitude, we simply called them theta amplitude and gamma amplitude.

### Coherence of Cross-frequency Coupling

We used coherence analysis to detect the modulation of phase to amplitude of the two band limited signals at each frequency band. In the literature, several different methods have been used to measure phase to amplitude modulation [13]–[15], [39]. In the current paper, we adopted the method proposed by Tort et al [39], which is outlined as follows:

- Separate the raw signal into two sets of band-pass filtered signals. The first set had centre frequencies from 2 Hz to 20 Hz, in 1 Hz steps with a 2 Hz bandwidth. This created a real-value band-pass filtered signal set 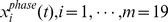. The second set of real-value band-pass filtered signals 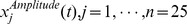 was created by filtering the raw signal with centre frequencies from 30 Hz to 70 Hz, in 2 Hz steps with a 4 Hz bandwidth.
- Extract the phase signals from 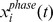 and the amplitude signals from 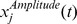, and apply a Hilbert Transform to both sets to generate complex-valued analytic band-passed signals. Denoted the phase sets as 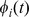 and the amplitude time series as 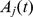,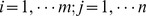.
- For each pair of signals 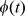 and 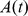, 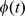 is binned into N intervals from 0 to 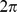 with 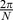 bin size (here N = 18), and the mean of amplitude of 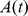 over each phase bin is calculated. We denoted by 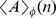 the mean amplitude at the nth phase bin.
- Normalize the mean amplitude to get a distribution-like function as the “amplitude distribution”:

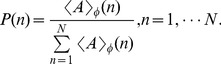
(9)And then calculate Kullback-Leiber (KL) distance of P from the uniform distribution U:
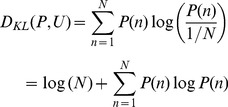
(10)

- The coherence of CFC between the ith phase signal 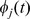 and the jth amplitude signal 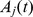 is then defined by dividing the above KL distance by log(N):

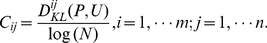
(11)We took the average of the above pair-wised coherence as the coherence of CFC between theta phase and gamma amplitude:
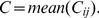
(12)


### Theta-phase Variation

The wavelet transform also provides phase information in the time-frequency domain. We applied the wavelet transform to the membrane potential of each EX neuron, and applied the Hilbert transform to take out the phase signal of the complex wavelet transform at 4–8 Hz frequency band for each EX neuron. We therefore obtained 100 different time series of phase signals. The time courses of phase signals of the 100 EX neurons shown in Figure 5C1 and Figure 6D were calculated by this method. To quantify the synchronization of theta-band phase between neurons, we calculated the variation of the phase at each fixed time and then averaged the variation over the whole period. This quantity is denoted as theta-phase variation to measure the concentration of theta-band phase. The smaller this value is, the more synchronized the phase of theta is between neurons.

## Supporting Information

Figure S1 **Stimulus-enhanced theta wave as well as CFC in a sparsely connected network with N_EX_ = 100, N_INf_ = 50, N_INs_ = 50, and the probability of connection p = 0.8.** (A) The firing behaviors of single INs, INf and EX neurons. The bottom trace is the firing pattern of 50 EX neurons. (B) The response of the LFP to a stimulus lasting 500 ms and correspondent time-dependent power spectrum of the LFP. (C) Coherence of CFC between theta phase and the gamma amplitude for the pre and during stimulus epochs.
(TIF)

Figure S2 **The corresponding figures in Fig.S1 for N_EX_ = 200, N_INf_ = 100, N_INs_ = 100, and the probability of connection p = 0.6.** The corresponding weights of connections are as follows: g_GAfe_ = 0.015; g_GAse_ = 0.06, g_NMee_ = 0.002, g_NMes_ = 0.0003, g_AMee_ = 0.007, g_AMef_ = 0.08, g_NMef_ = 0.003, g_GAff_ = 0.08, g_GAfs_ = 0.0, g_GAsf_ = 0.1, g_AMes_ = 0.005, g_GAss_ = 0.08.
(TIF)

Figure S3 **Effects of increasing only NMDA receptor (gNMee and gNMes) strengths in a sparse network on (A) theta and gamma amplitude, (B) the coherence of CFC between theta phase and gamma amplitude and (C) the variation of theta-band phase.**
(TIF)

Figure S4 **Dependence of theta amplitude and gamma amplitude (A), the coherence of CFC (B) and the variation of theta-band phase (C) on the EX-to-EX connection mediated by NMDAR and the Ins-to-EX connection mediated by slow GABAA receptors.**
(TIF)

Figure S5 **Increasing theta-gamma coupling without a corresponding change in theta amplitude by appropriately increasing the couplings g_GAfe_, g_GAff_ and g_GAsf_ together.** In (A1–C1), to mimic the learning effects, the values of the couplings (gGAfe, gGAff,gGAsf) are chosen as: (0.007, 0.03,0.02) for 1st, (0.01,0.05,0.02) for 2nd, (0.015,0.06,0.035) for 3rd, and (0.02, 0.07, 0.04) for 4th. In (A2–C2), the theta and gamma amplitudes, the coherence of CFC and the phase variation are plotted vs. the stimulus strength. The black curve corresponds to before learning with (gGAfe, gGAff, gGAsf) = (0.007,0.03,0.02), the pink one corresponds to after learning with (gGAfe, gGAff, gGAsf) = (0.015,0.06,0.03).
(TIF)

Text S1 **Supporting material: Results in a sparse network.**
(DOCX)
