# Supplementary figures and images for: A Computational Study on Altered Theta-Gamma Coupling during Learning and Phase Coding
Source: PLoS One. 2012 Jun 21;7(6):e36472. doi: 10.1371/journal.pone.0036472 (PMC3380897; doi:10.1371/journal.pone.0036472)

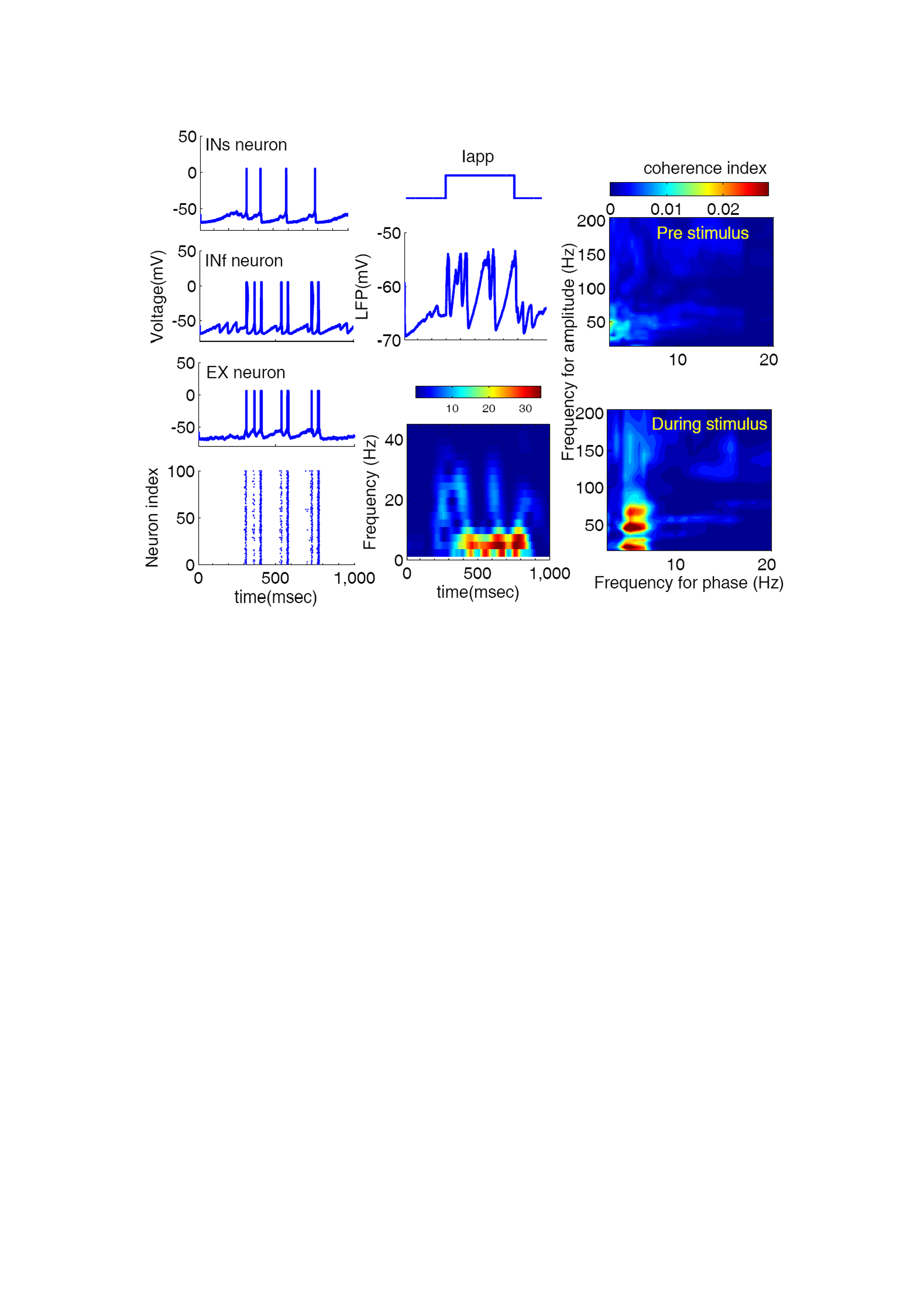

Supplement: Figure S1 — Stimulus-enhanced theta wave as well as CFC in a sparsely connected network with NEX = 100, NINf = 50, NINs = 50, and the probability of connection p = 0.8. (A) The firing behaviors of single INs, INf and EX neurons. The bottom trace is the firing pattern of 50 EX neurons. (B) The response of the LFP to a stimulus lasting 500 ms and correspondent time-dependent power spectrum of the LFP. (C) Coherence of CFC between theta phase and the gamma amplitude for the pre and during stimulus epochs. (TIF) [file pone.0036472.s001.tif]

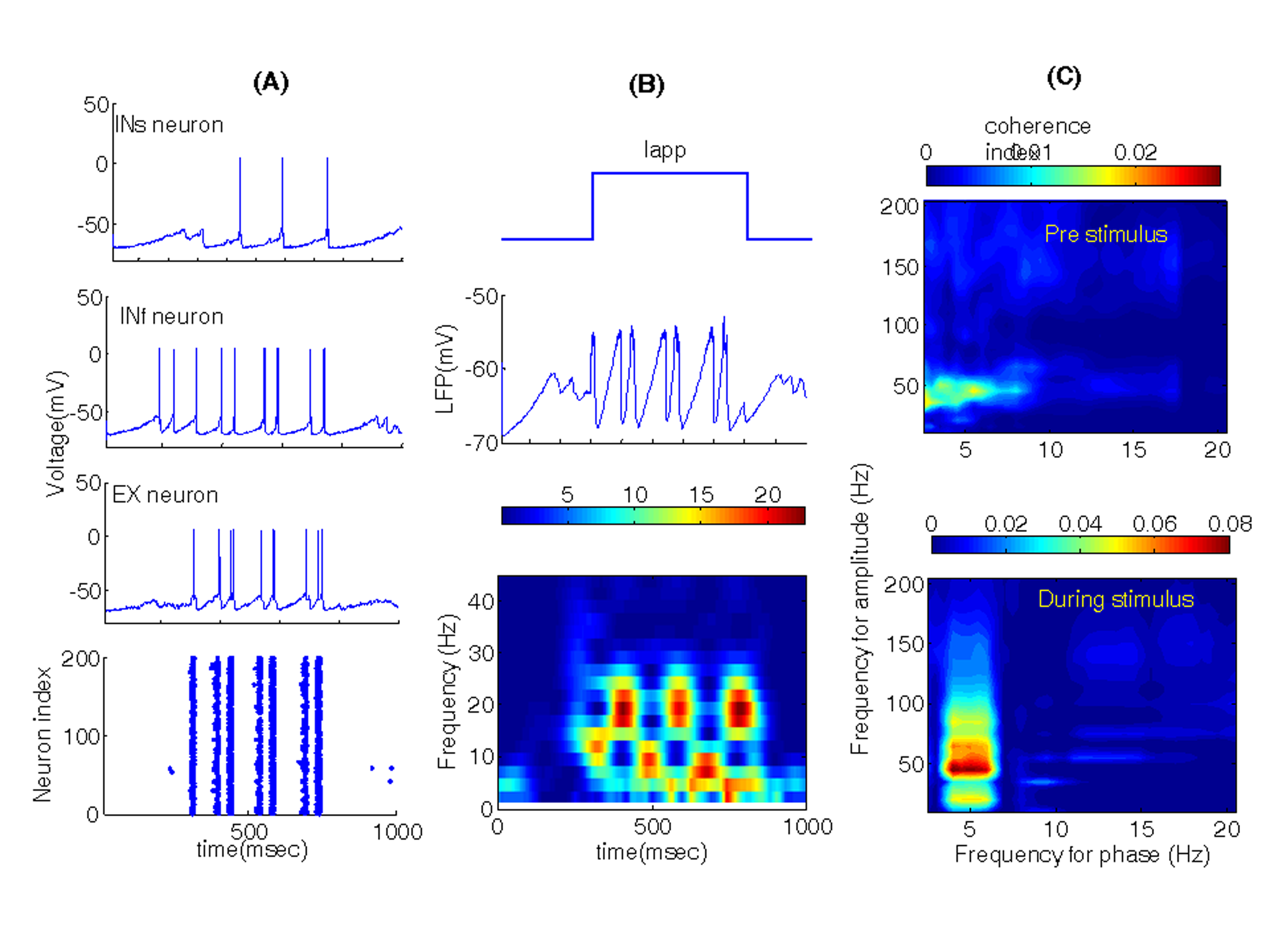

Supplement: Figure S2 — The corresponding figures in Fig.S1 for NEX = 200, NINf = 100, NINs = 100, and the probability of connection p = 0.6. The corresponding weights of connections are as follows: gGAfe = 0.015; gGAse = 0.06, gNMee = 0.002, gNMes = 0.0003, gAMee = 0.007, gAMef = 0.08, gNMef = 0.003, gGAff = 0.08, gGAfs = 0.0, gGAsf = 0.1, gAMes = 0.005, gGAss = 0.08. (TIF) [file pone.0036472.s002.tif]

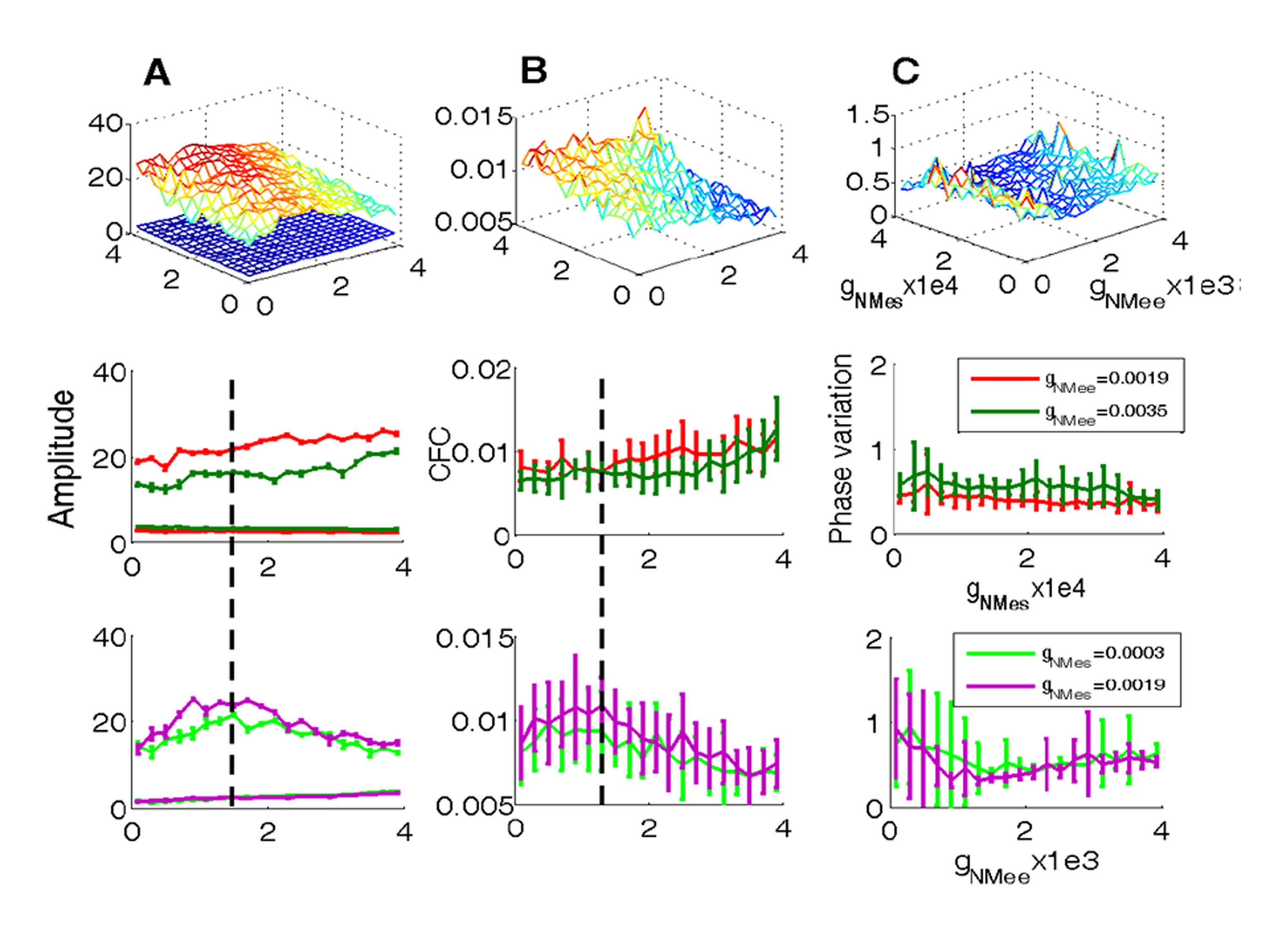

Supplement: Figure S3 — Effects of increasing only NMDA receptor (gNMee and gNMes) strengths in a sparse network on (A) theta and gamma amplitude, (B) the coherence of CFC between theta phase and gamma amplitude and (C) the variation of theta-band phase. (TIF) [file pone.0036472.s003.tif]

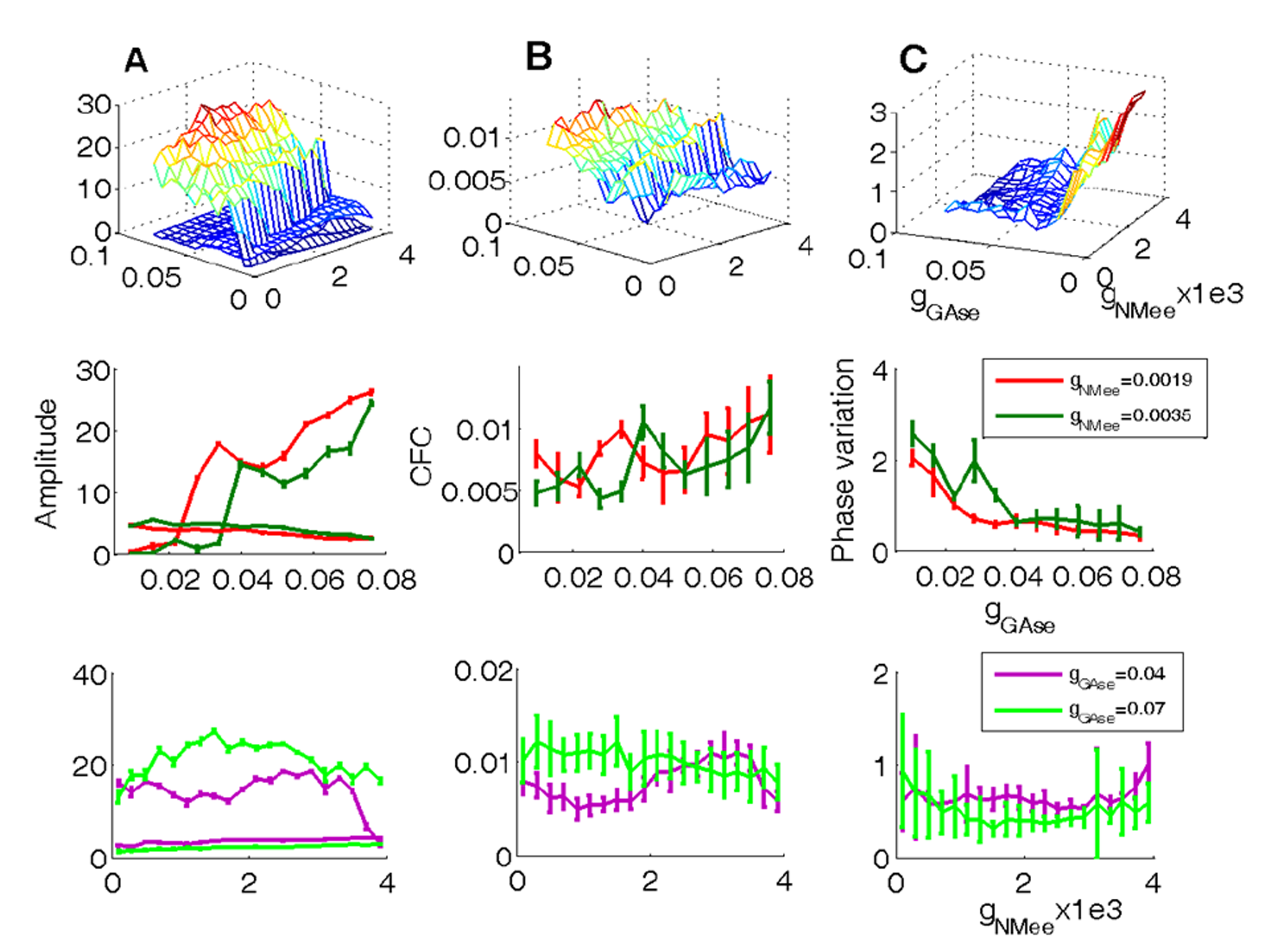

Supplement: Figure S4 — Dependence of theta amplitude and gamma amplitude (A), the coherence of CFC (B) and the variation of theta-band phase (C) on the EX-to-EX connection mediated by NMDAR and the Ins-to-EX connection mediated by slow GABAA receptors. (TIF) [file pone.0036472.s004.tif]

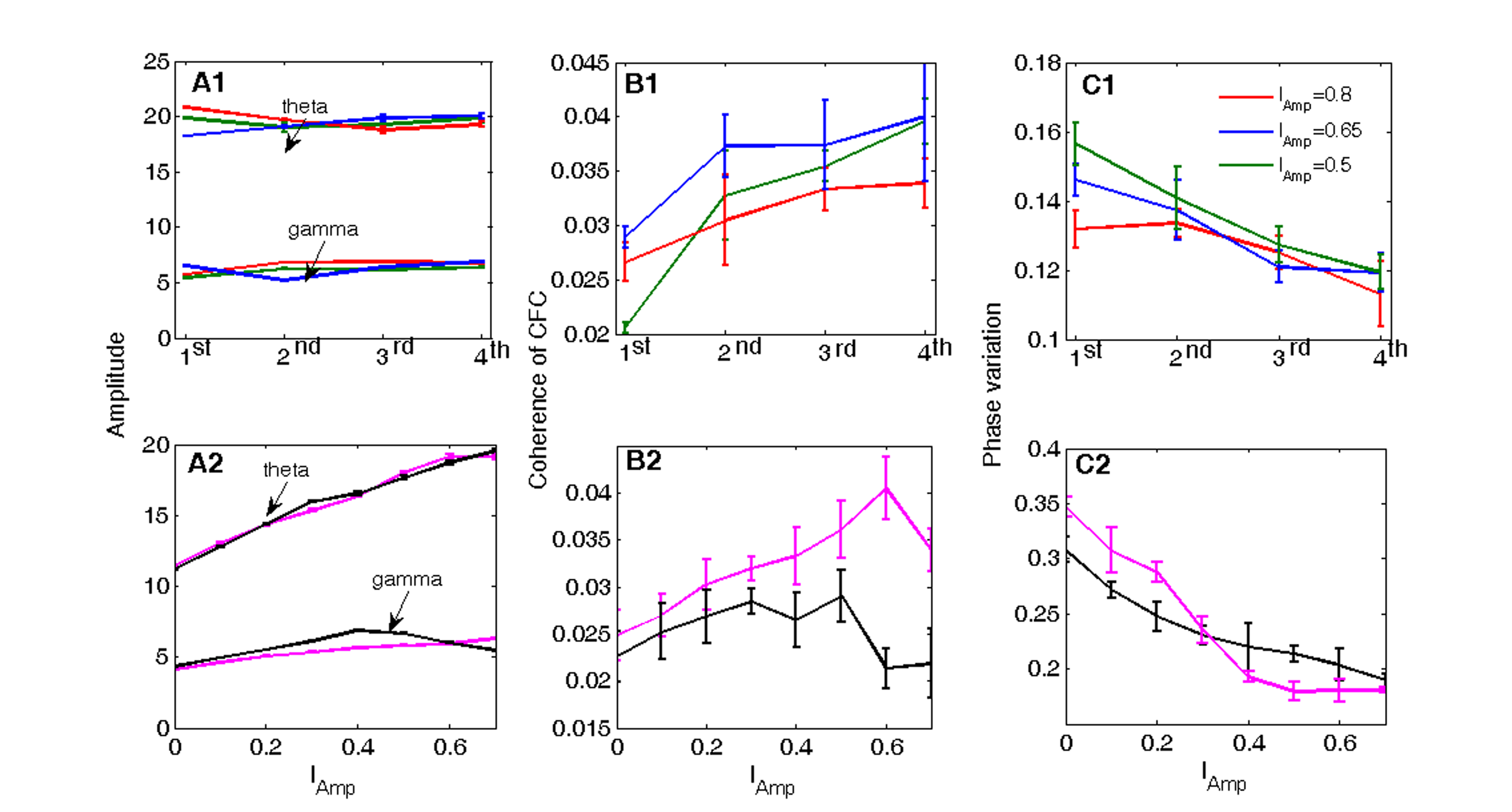

Supplement: Figure S5 — Increasing theta-gamma coupling without a corresponding change in theta amplitude by appropriately increasing the couplings gGAfe, gGAff and gGAsf together. In (A1–C1), to mimic the learning effects, the values of the couplings (gGAfe, gGAff,gGAsf) are chosen as: (0.007, 0.03,0.02) for 1st, (0.01,0.05,0.02) for 2nd, (0.015,0.06,0.035) for 3rd, and (0.02, 0.07, 0.04) for 4th. In (A2–C2), the theta and gamma amplitudes, the coherence of CFC and the phase variation are plotted vs. the stimulus strength. The black curve corresponds to before learning with (gGAfe, gGAff, gGAsf) = (0.007,0.03,0.02), the pink one corresponds to after learning with (gGAfe, gGAff, gGAsf) = (0.015,0.06,0.03). (TIF) [file pone.0036472.s005.tif]
