# Supplementary material for: A Computational Study on Altered Theta-Gamma Coupling during Learning and Phase Coding
Source: PLoS One. 2012 Jun 21;7(6):e36472. doi: 10.1371/journal.pone.0036472 (PMC3380897; doi:10.1371/journal.pone.0036472)
Supplement: Text S1 — Supporting material: Results in a sparse network. (DOCX) [file pone.0036472.s006.docx]

**Supporting material: Results in a sparse network**

1. **Stimulus-enhanced theta/gamma oscillation in a sparse network**

The results in the main text are obtained using an all-to-all connected network. We have additionally investigated whether our results can also be generated using a more physiological sparsely connected network. Here the sparseness of the network is realized by randomly assigning the coupling between neurons and the probability that a pair of neurons are connected in either direction is p=0.8. The network size is still the same as in the all-to-all network i.e. NR=100, Nf=50, Ns=50. The maximum synaptic conductances are slightly modified as:

$$g{}_{\mathrm{GAfe}}= 0.01; g_{\mathrm{GAse}} = 0.06; g_{\mathrm{NMee}} = 0.001; g_{\mathrm{NMes}} = 0.0001;$$

$$g_{\mathrm{AMee}} = 0.01; g_{\mathrm{AMef}}= 0.05; g_{\mathrm{NMef}} = 0.005; g_{\mathrm{GAff}}= 0.08;$$

$g_{\mathrm{GAsf}} = 0.05; g_{\mathrm{AMes}}= 0.005; g_{\mathrm{GAss}} = 0.08;$

Figure S1 shows that for a sparse network, the theta-gamma dual oscillations can be generated by by utilizing a combination of fast and slow-type GABAA receptor interneurons. Similar to the case in an all-to-all network, these theta/gamma dual oscillations in a sparse arrangement also show stimulus-enhanced theta power with a weak gamma-band power. As for the cross frequency coupling (CFC) between theta phase and gamma amplitude, the result is also similar to the all-to-all network, i.e., the coherence of the CFC is increased during the stimulus compared with the pre-stimulus period. Figure S2 shows that when the network size increases to *N*_EX_ =200, *N*_INf_ =100, *N*_INs_ =100, the coupling probability can be reduced to p=0.6 and all results still hold true. We anticipate that the probability of connectivity could be further reduced with an increased network size.

1. **Different synaptic mechanism for learning-related changes in the theta amplitude and the CFC**

In Figure S3 and Figure S4, we show the dependence of the theta and gamma amplitudes, the coherence of the CFC between theta phase and gamma amplitude, the variation of theta-band phase on different types of synaptic connections in a sparse network. Figure S3 shows the dependence on $g_{\mathrm{NMee}}$ and $g_{\mathrm{NMes}}$ conductances. Over a small range the effects of these two parameters on theta amplitude have a slight difference to results shown using an all-to-all network shown in Figure 4 in the main text. With the sparse network both theta amplitude and the coherence of CFC can be increased by moderately increasing the conductance $g_{\mathrm{NMee}}$, however further increases result in a decrease of theta amplitude and the CFC. Changing the conductance $g_{\mathrm{NMes}}$, does not have a significant effect in increasing theta amplitude, but can lead to an increase of the CFC strength. In Figure S4 effects of altering $g_{\mathrm{NMee}}$ and $g_{\mathrm{GAse}}$ conductances can be seen. Increasing $g_{\mathrm{NMee}}$ and $g_{\mathrm{GAse}}$ can lead to a significant and more robust increase of both theta amplitude and the coherence of CFC, and the theta-band phase becomes more synchronized among neurons. These results are consistent with those shown in Figure 5 in the main text for an all-to-all connected network.

In conclusion in a sparsened network: i). both theta amplitude and the CFC can also be increased by moderately increasing the EX-to-EX connection and the EX-to-INs connection mediated by NMDA receptors, but only in a narrow parameter range. ii). Co-ordinately increasing the EX-to-EX connection mediated by NMDA receptors and the INs-to- EX connection mediated by slow GABA_A_ receptors can also significantly and robustly increase theta amplitude and the CFC as well as the synchronization of theta-band phase among neurons.
